# Dr. Blackburn, on Light

**Published:** 1800-06

**Authors:** 


### *Dr. Blackburn, on Light.*

WE have to lament, that Dr. Blackburn has been prevented by his avocations during the laft twelve months, and at prefent has not fufficient leifure to arrange the proofs and reafoning upon which his conclufions are founded: he has, however, permitted us to lay before the public, the refults of his inveftigation in the abftract, viz.

1ft. Light is a compound refulting from a peculiar combination of caloric and oxygen.

2dly. In all thofe phenomena which have given occafion to the idea that light is identical with, or a modification of, caloric, the manifeftation of this latter principle is to be referred to the difunion of the conftituent principles of light. The caloric, therefore, which light fo frequently exhibits, is the refult of its decompofition.

3dly. The phenomena of colours are to be afcribed to the different qualities of light, as containing caloric and oxygen in different proportions. Thefe different proportions manifeft themfelves in the circumftances both of the decompofition and the formation of varioufly coloured light.

4th. The feparation of light by the prifm, is to be regarded as a chemical decompofition, not a phyfical or mechanical divifion of light.

5th. The changes which take place in the colours of different fubftances, as of plants during the procefs of vegetation; of metals during that of oxydation, are referable to correfpondent changes, which theſe ſubſtances experience in their chemical action upon light.

6th. The evaneſcence, or, as it is frequently termed, the abſorption of light, is owing to the complete reſolution of the compound into its conſtituent parts.

